# Supplementary material for: SyNDI: synchronous network data integration framework
Source: BMC Bioinformatics. 2018 Nov 6;19:403. doi: 10.1186/s12859-018-2426-5 (PMC6219086; doi:10.1186/s12859-018-2426-5)
Supplement: Supplementary file 5 — Files of the example presented in Additional file 4. This additional file is a zip package that contains all files used in Additional file 4. Detailed descriptions of each of these files are presented in a README file included in this zip package. (GZ 30411 kb) [file 12859_2018_2426_MOESM5_ESM.gz › SyNDITools/Meme2Fimo/meme_4.9.0_4.tar.gz!/meme_4.9.0/doc/glam2_man.html]

GLAM2 Manual


# GLAM2 Manual

**Summary:** GLAM2 is a software package for finding
*motifs* in sequences, typically amino-acid or nucleotide
sequences. A motif is a re-occurring sequence pattern: typical examples
are the TATA box and the CAAX prenylation motif. The main innovation of
GLAM2 is that it allows insertions and deletions in motifs.

**Family:** GLAM2 is a child of GLAM and a sibling of A-GLAM.
If you are looking for nucleotide motifs without insertions or
deletions, A-GLAM is probably better. 'GLAM' used to stand for Gapless
Local Alignment of Multiple sequences, but it now stands for Gapped
Local Alignment of Motifs.

Inventory: The package includes these programs:

- **glam2** - for discovering motifs shared by a
  set of sequences.
- **glam2scan** - for finding matches, in a
  sequence database, to a motif discovered by glam2.
- **glam2format** - for converting glam2 motifs to
  standard alignment formats.
- **glam2mask** - for masking glam2 motifs out of
  sequences, so that weaker motifs can be found.
- **purge** - for removing highly similar members
  of a set of sequences.

These are *command line* programs, so you need a basic
familiarity with running things from your computer's command line in
order to use them. (In Mac OS X, you want Applications -> Utilities ->
Terminal.)

**About:** GLAM2 was developed by Martin C Frith, working at the Computational Biology
Research Center in Tokyo, and Timothy L Bailey,
working at the Institute for
Molecular Bioscience in Brisbane. The source code and documentation are
hereby released into the public domain. If you use GLAM2, please cite: MC
Frith, NFW Saunders, B Kobe, TL Bailey (2008) Discovering sequence
motifs with arbitrary insertions and deletions, PLoS Computational
Biology, **4**(5), e1000071, 2008. .

**Contact:** Questions and comments are welcome.
Email: glam2(at-mark)imb.uq.edu.au. We would especially like to hear
about any errors when installing or running the software (e.g.
segmentation faults, assertion failures) so that we can fix them. Please
send the exact text of the error message, and the exact inputs that
produce the error.

## Contents

- Installation - how to install
  GLAM2 on your computer.
- glam2 tutorial - all you need to
  know for typical usage of glam2.
- glam2 reference - all of glam2's
  options.
- glam2scan manual
- glam2format manual
- glam2mask manual
- purge manual
- Alphabets
- Dirichlet mixtures
- GLAM2 methods (PDF) -
  mathematical description of how GLAM2 works.
